# Safety and Immunogenicity of a Revaccination With a Respiratory Syncytial Virus Prefusion F Vaccine in Older Adults: A Phase 2b Study

**DOI:** 10.1093/infdis/jiad321

**Published:** 2023-09-12

**Authors:** Isabel Leroux-Roels, Marc Van Ranst, Corinne Vandermeulen, Carline Vanden Abeele, Nathalie De Schrevel, Bruno Salaun, Céline Verheust, Marie-Pierre David, Shady Kotb, Veronica Hulstrøm

**Affiliations:** Centre for Vaccinology, Ghent University and Ghent University Hospital Ghent, Belgium; Rega Institute for Medical Research, Katholieke Universiteit Leuven, Leuven, Belgium; Leuven University Vaccinology Centre, Katholieke Universiteit Leuven, Leuven, Belgium; GSK, Wavre, Belgium; GSK, Rixensart, Belgium; GSK, Rixensart, Belgium; GSK, Wavre, Belgium; GSK, Wavre, Belgium; GSK, Wavre, Belgium; GSK, Wavre, Belgium

**Keywords:** AS01E, RSV neutralizing titers, RSV vaccine, RSVPreF3, cell-mediated immunity, respiratory syncytial virus

## Abstract

**Background:**

In the previous (parent) study, 2 doses of different formulations of an investigational vaccine against respiratory syncytial virus (RSVPreF3 OA) were well tolerated and immunogenic in older adults. This multicenter phase 2b extension study assessed safety and immunogenicity of a revaccination (third) dose of the 120 μg RSVPreF3-AS01_E_ formulation.

**Methods:**

In total, 122 older adults (60–80 years), previously vaccinated with 2 doses of RSVPreF3-AS01_E_ formulations (containing 30, 60, or 120 μg RSVPreF3 antigen), received an additional 120 μg RSVPreF3-AS01_E_ dose 18 months after dose 2. Vaccine safety was evaluated in all participants up to 6 months and immunogenicity in participants who received 120 μg RSVPreF3-AS01_E_ doses until 1 month after dose 3.

**Results:**

Similar to the parent study, mostly mild-to-moderate solicited adverse events and no vaccine-related serious adverse events or potential immune-mediated disorders were reported. Neutralizing titers and cell-mediated immune responses persisted for 18 months after 2-dose vaccination. Dose 3 increased RSV-specific neutralizing titers against RSV-A and RSV-B and median CD4^+^ T-cell frequencies. After dose 3, RSV-specific neutralizing titers but not CD4^+^ T-cell frequencies were below levels detected 1 month after dose 1.

**Conclusions:**

Revaccination with 120 μg RSVPreF3-AS01_E_ 18 months after dose 2 is well tolerated and immunogenic in older adults.

**Clinical Trials Registration:**

NCT04657198; EudraCT, 2020-000692-21.

## Teaser

This phase 2b extension study demonstrated an acceptable safety profile and robust immunogenicity of a third, revaccination, dose of an adjuvanted vaccine against respiratory syncytial virus administered 18 months after the second dose in older adults aged ≥60 years.

Respiratory syncytial virus (RSV) is a contagious seasonal virus causing respiratory tract infections in people of all ages [1, 2]. There are 2 main antigenic subtypes, RSV-A and RSV-B [1]. The subtypes are cocirculating with alternating predominance across seasons, with a varying pattern [1, 2].

RSV infections usually resolve without complications or sequelae in immune-competent persons [3]. However, in older adults (OAs) aged ≥60 years, RSV can cause more serious respiratory illnesses (including lower respiratory tract disease) [3], especially in people with underlying medical conditions or those who are immunocompromised [4, 5]. In OAs, RSV infections thus lead to a significant disease burden [6], which was underestimated for a long time [7–9]. According to a recent systematic review of data from high-income countries, the calculated pooled estimates of RSV acute respiratory infections in OAs aged ≥60 years were 1.62% (95% confidence interval [CI], .84%–3.08%) for attack rate, 0.15% (95% CI, .09%–.22%) for hospitalization rate, and 7.13% (95% CI, 5.40%–9.36%) for in-hospital case fatality rate [8]. Based on the described values and using the 2019 census data, the same review estimated that about 5 million cases of acute respiratory tract infection, half a million hospitalizations, and 33 000 in-hospital deaths of OAs could be attributed to RSV in 2019 [8].

The severity of RSV-associated disease in OAs has been ascribed to waning humoral and cellular immune responses (immunosenescence) that were induced by previous RSV infections [10–15]. A protective immune response against RSV is orchestrated by antibodies (eg, immunoglobulin A [IgA] and IgG, neutralizing antibodies [nAb]), and lymphocytes (both cluster-of-differentiation-4-expressing [CD4^+^] and CD8^+^ T cells) that produce a variety of cytokines such as interleukins (ILs) and interferons (IFNs), resulting in viral clearance and protection [14]. The RSV-specific immunity obtained after infection is not long lasting and, even though most people have some level of postinfection immunity, this does not prevent subsequent RSV infections. Due to a higher disease burden in the vulnerable OA population, the waning immune responses lead to an increased risk for more severe disease in OAs. Thus, approaches to overcome waning immunity (eg, vaccination) can help avoid serious RSV-associated disease in OAs [16].

Several vaccines based on the prefusion conformation of RSV fusion protein (PreF) and using different delivery systems were recently evaluated in clinical studies [17–26]. The RSV vaccine investigated in this study is based on PreF stabilized in its trimeric conformation (RSVPreF3) as the main antigen, and adjuvanted with AS01_E_ [18, 27]. In a previous phase 1/2 study (hereafter referred to as the parent study), different formulations of the RSVPreF3-based vaccine were administered 2 months apart to OAs aged 60–80 years [18]. The vaccine formulation containing 120 µg of RSVPreF3 and adjuvanted with AS01_E_ (hereafter referred to as 120 μg RSVPreF3-AS01_E_ or RSVPreF3 OA) was selected for further clinical development, because it most potently induced humoral and cellular RSV-specific immune responses while retaining an acceptable safety profile in the parent study [18]. An ongoing vaccine efficacy study demonstrated a consistently high efficacy (point estimate of 82.6% with a 96.95% CI, 57.9%–94.1%) for the 120 μg RSVPreF3-AS01_E_ formulation against RSV-related lower respiratory tract disease in OAs aged ≥60 years, thus meeting the primary study end point [27, 28]. The 120 μg RSVPreF3-AS01_E_ formulation has then been licensed for use in OAs in the United States and European Union [29].

The overall objective of the present extension study was to evaluate the safety, reactogenicity, and immunogenicity of the selected 120 μg RSVPreF3-AS01_E_ formulation, administered as the third vaccine dose (dose 3) 18 months after dose 2 (month 20 [M20]), in participants who had received 2 doses of the AS01_E_-adjuvanted formulations containing 30, 60, or 120 μg of RSVPreF3 (ie, 30 μg RSVPreF3-AS01_E_, 60 μg RSVPreF3-AS01_E_, or 120 μg RSVPreF3-AS01_E_) in the parent study.

## METHODS

This phase 2b extension study (NCT04657198) was conducted in accordance with the Declaration of Helsinki and the International Council for Harmonization requirements. The study was approved by institutional ethics committees. The participating OAs were enrolled at 7 centers in the United States, and 3 centers in Belgium. The study was open label as all participants received the same 120 μg RSVPreF3-AS01_E_ vaccine formulation.

### Study Vaccine

The RSVPreF3 vaccine formulations have been described in detail [18]. In this extension study, only the 120 μg RSVPreF3-AS01_E_ formulation was administered as the third dose.

### Study Participants and Procedures

Eligible participants were healthy men and women who had received the 30-, 60-, or 120 μg RSVPreF3-AS01_E_ formulation in the parent study [18]. Participants needed to be able and willing to comply with protocol requirements (as determined by investigator), and to have provided written informed consent prior to any study-specific procedures. Deviations from inclusion criteria were not allowed. Inclusion and exclusion criteria are listed in the Supplementary Material.

**Figure 1.**
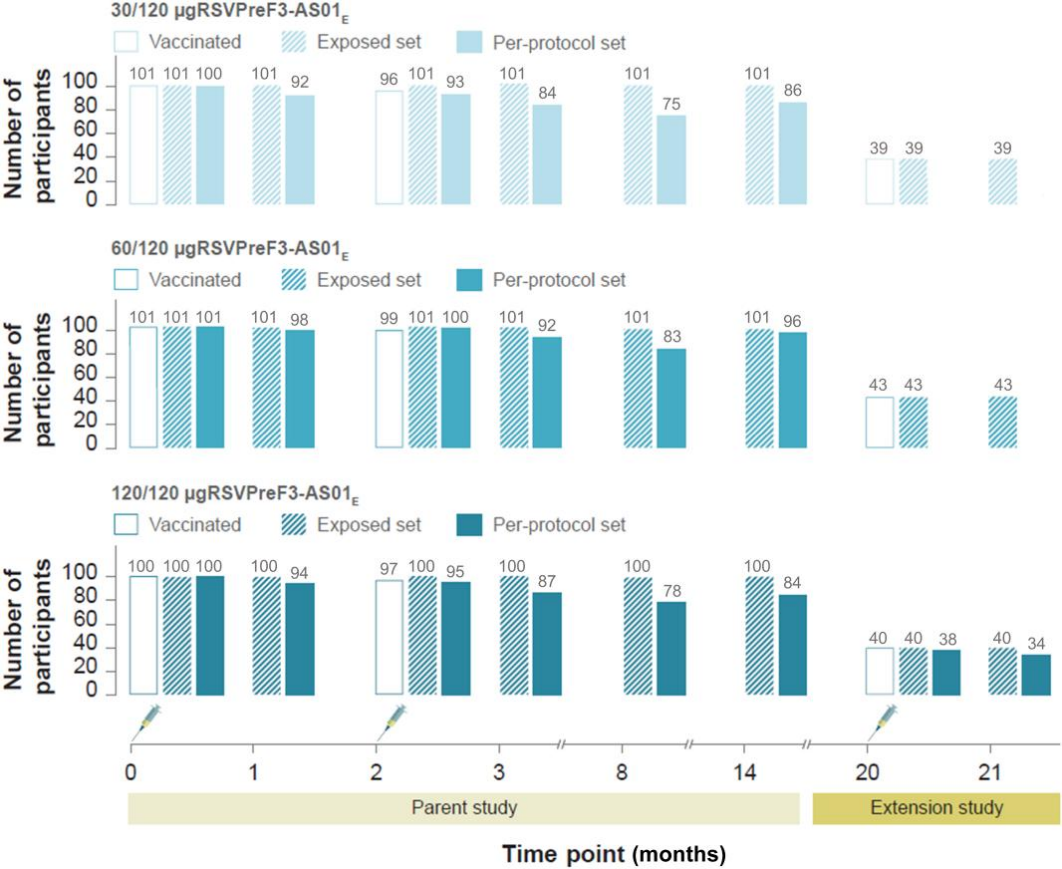
Overview of parent and extension study designs. The parent study design and data have been published [18]. Syringe symbols represent vaccination. Time points 0, 1, 2, 3, 8, 14, 20, and 21 indicate study time points at M0 (day 1, dose 1 vaccination), M1 (day 31, 1 month after dose 1), M2 (day 61, dose 2 vaccination), M3 (day 91, 1 month after dose 2), M8, M14, M20 (dose 3 vaccination), and M21 (1 month after dose 3). Participants received 2 doses of the AS01_E_-adjuvanted vaccine formulation with 30, 60, or 120 μg of RSVPreF3 antigen in the parent study and a third dose of the AS01_E_-adjuvanted vaccine formulation containing 120 μg of RSVPreF3 antigen in the extension study, indicated by 30/120-, 60/120-, and 120/120 μg RSVPreF3-AS01_E_. Abbreviations: AS01_E_, adjuvant system; M, month; RSVPreF3, prefusion conformation of the respiratory syncytial virus fusion (F) protein.

In the parent study [18], the OA participants were randomized to receive 2 doses of a given vaccine formulation or placebo on day 1 (also denoted as M0 time point) and day 61 (M2) (Figure 1). The follow-up period for OAs was up to 1 year after the second vaccination (M14). In this extension study, all OA recipients of the AS01_E_-adjuvanted formulations were invited to receive the third vaccine dose containing the 120 μg RSVPreF3-AS01_E_ formulation at M20. Follow-up time was 6 months after dose 3 (M26). All vaccines were administered intramuscularly, into the deltoid region of the nondominant arm.

Because the present manuscript refers to findings of both the parent and extension studies, the timeline details are provided here for ease of reference. Time points M0–M14 refer to the parent study and included M0 (day 1, baseline, dose 1 administration), M1 (day 31, 1 month after dose 1), M2 (day 61, dose 2 administration), M3 (day 91, 1 month after dose 2), M8, and M14. This extension study includes time points M20 (dose 3 administration), M21 (1 month after dose 3), and M26 (end of study, 6 months after dose 3) (Figure 1).

Participant groups were named 30/120 μg RSVPreF3-AS01_E_, 60/120 μg RSVPreF3-AS01_E_, and 120/120 μg RSVPreF3-AS01_E_ according to the RSVPreF3-based vaccine formulations received in both studies (eg, the group 30/120 μg RSVPreF3-AS01_E_ received 2 doses of the 30 μg RSVPreF3-AS01_E_ formulation in the parent study and the 120 μg RSVPreF3-AS01_E_ formulation in this extension study).

Occurrence of adverse events (AEs) was recorded for all participants in the following periods: 4 days after dose 3 for solicited AEs (administration site [pain, redness, swelling] and systemic [fever]) AEs, 30 days after dose 3 for unsolicited AEs, and up to 6 months after dose 3 for AEs leading to study withdrawal, serious AEs (SAEs) and potential immune-mediated disorders (pIMDs).

Blood samples for evaluation of humoral (approximately 20 mL) and cell-mediated immune (CMI) (approximately 25 mL) responses were collected at M20 and M21 time points only from participants in the 120/120 μg RSVPreF3-AS01_E_ group. Neutralizing titers against RSV-A and RSV-B were measured by neutralization assays, and RSVPreF3-specific IgG concentrations were determined using an in-house enzyme-linked immunosorbent assay (ELISA) [18]. Frequencies of RSVPreF3-specific CD4^+^ and CD8^+^ T cells were evaluated using intracellular cytokine staining on peripheral blood mononuclear cells [18].

### Study Objectives and End Points

Primary safety objectives were to evaluate the vaccine safety and reactogenicity in all participants up to 1 month after dose 3 in terms of occurrence of solicited AEs up to 4 days, and unsolicited AEs, SAEs, and pIMDs up to 30 days after dose 3. The primary immunogenicity objective was to evaluate humoral immune responses in the 120/120 μg RSVPreF3-AS01_E_ group of participants in terms of neutralizing titers against RSV-A and RSV-B up to 1 month after dose 3 (M21).

The secondary safety objective was to determine the safety of dose 3 in all participants until study end (ie, 6 months after dose 3) in terms of occurrence of SAEs and pIMDs. The secondary immunogenicity objective was to evaluate the humoral response in terms of RSVPreF3-specific IgG concentration and CMI response in terms of frequency of RSVPreF3-specific CD4^+^ T cells expressing at least 2 markers (among IL-2, CD40 ligand [CD40L], tumor necrosis factor-α [TNF-α] and IFN-γ) in the 120/120 μg RSVPreF3-AS01_E_ group of participants up to M21. Tertiary study objectives and end points are described in the Supplementary Material.

### Statistical Analyses

Sample size for the parent study was previously presented in detail [18], and no additional estimations were done for this extension study. Analysis sets included enrolled set (participants who provided their informed consent to participate in the study), exposed set (participants who received dose 3), and per-protocol set (participants who received dose 3 with available immunogenicity data and without important protocol deviations including those leading to study exclusion [see Supplementary Material]). Safety was assessed on the exposed set, while immunogenicity was evaluated on the per-protocol set.

All data were analyzed using descriptive statistics. Categorical data were tabulated as the number and percentage of participants, while continuous data were described/plotted as mean with 95% CI or median with range (minimum and maximum).

The geometric mean titers/concentrations (GMTs/GMCs) were computed as the antilogarithm of the arithmetic mean of the log_10_ transformed titers/concentrations. Cutoff or lower limit of quantification (LLOQ) values for immunogenicity assays were: 18 estimated dilution 60 (ED60) (RSV-A nAb GMT), 30 ED60 (RSV-B nAb GMT), 25 ELISA units/mL (RSVPreF3-specific IgG GMC), and 590/10^6^ cells (CD4^+^ T-cell frequencies). Titers/concentrations below the assay cutoff were given an arbitrary value of half the assay cutoff, while those above the assay's upper limit of quantification (ULOQ) were assigned the ULOQ value. For calculations of the fold change in frequencies of CD4^+^ T cells expressing at least 2 markers, frequencies below the LLOQ were imputed to the LLOQ value. Missing or nonevaluable measurements were not replaced.

## RESULTS

### Demographic and Baseline Characteristics of Study Participants

In the parent study, 1005 OA participants received at least 1 vaccine/placebo dose [18]. Of those, 302 received 1 and 291 received 2 doses of an RSVPreF3-AS01_E_ vaccine formulation (Figure 1) [18].

**Figure 2.**
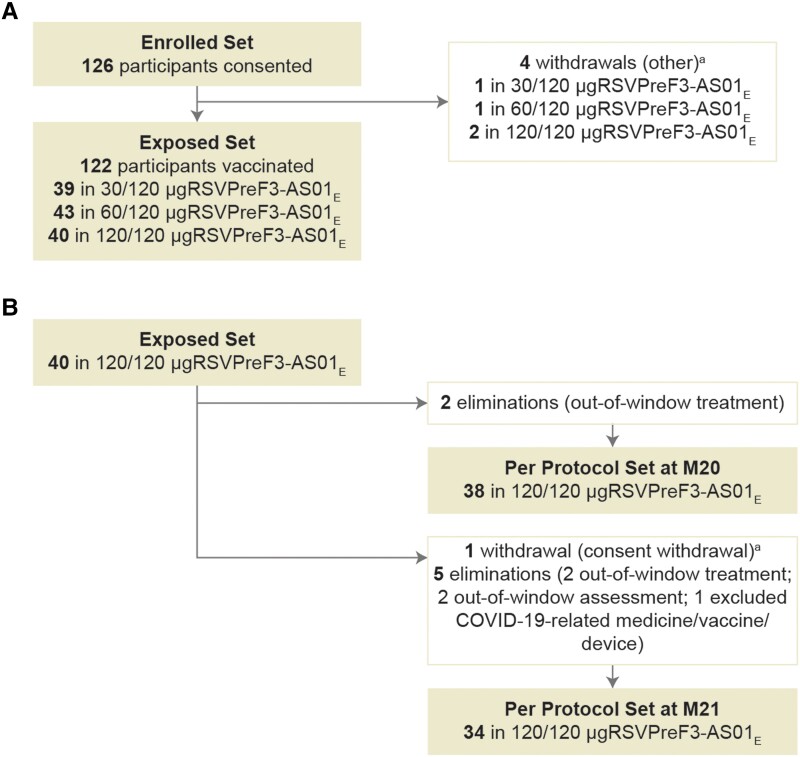
Participant flow chart with (*A*) reasons for withdrawal and elimination from the exposed set and (*B*) per-protocol set. ^a^Participant withdrawal (including consent withdrawal) was due to a reason other than an adverse event and/or solicited adverse event, migration from study area, loss to follow-up, or sponsor study termination. Participants received 2 doses of the AS01_E_-adjuvanted vaccine formulation with 30, 60, or 120 μg of RSVPreF3 antigen in the parent study and a third dose of the AS01_E_-adjuvanted vaccine formulation containing 120 μg of RSVPreF3 antigen in the extension study, indicated by 30/120-, 60/120-, and 120/120 μg RSVPreF3-AS01_E_. M20 and M21 indicate study time points at month 20 (dose 3 vaccination) and month 21 (1 month after dose 3) in the extension study. Abbreviations: COVID-19, coronavirus disease 2019; M, month; RSVPreF3, prefusion conformation of the respiratory syncytial virus fusion (F) protein.

In this study, conducted between December 2020 and October 2021, 122 OA participants (39 in the 30/120 μg RSVPreF3-AS01_E_ group, 43 in the 60/120 μg RSVPreF3-AS01_E_ group, and 40 in the 120/120 μg RSVPreF3-AS01_E_ group) were included in the exposed set (Figure 1 and Figure 2). The per-protocol set included 38 (95.0%) and 34 (85.0%) of 120/120 μg RSVPreF3-AS01_E_ participants at M20 and M21 (Figure 2). The present study enrolled 72 (59.0%) female participants, and most participants were White (117, 95.9%) and of non-Hispanic or Latino ethnicity (121, 99.2%) (Table 1).

**Table 1.** Demographic Characteristics of OA Participants in the Parent^a^ and Extension Studies, Exposed Set

OA Participants, 60─80 y,	Parent Study				Extension Study			
Group	30 μg AS01_E_	60 μg AS01_E_	120 μg AS01_E_	Total	30/120 μg RSVPreF3-AS01_E_	60/120 μg RSVPreF3-AS01_E_	120/120 μg RSVPreF3-AS01_E_	Total
No. of participants	101	101	100	1005	39	43	40	122
Age at first vaccination, y								
Mean (SD)	67.8 (5.1)	67.1 (5.6)	67.6 (5.2)	67.6 (5.2)	69.1 (5.3)	66.6 (5.6)	68.3 (5.4)	68.0 (5.5)
Median (min–max)	67.0 (60.0–80.0)	66.0 (60.0–79.0)	67.0 (60.0–80.0)	67.0 (60.0–80.0)	69.0 (61.0–78.0)	64.0 (60.0–79.0)	68.5 (60.0–79.0)	67.0 (60.0–79.0)
Age category, y, No. (%)								
60–69	67 (66.3)	66 (65.3)	64 (64.0)	660 (65.7)	22 (56.4)	28 (65.1)	21 (52.5)	71 (58.2)
70–80	34 (33.7)	35 (34.7)	36 (36.0)	345 (34.3)	17 (43.6)	15 (34.9)	19 (47.5)	51 (41.8)
Sex, No. (%) Female	58 (57.4)	57 (56.4)	57 (57.0)	573 (57.0)	21 (53.8)	24 (55.8)	27 (67.5)	72 (59.0)
Ethnicity, No. (%) not Hispanic or Latino	98 (97.0)	98 (97.0)	97 (97.0)	969 (96.4)	39 (100.0)	42 (97.7)	40 (100.0)	121 (99.2)
Race, No. (%)								
White	88 (87.1)	94 (93.1)	93 (93.0)	927 (92.2)	37 (94.9)	42 (97.7)	38 (95.0)	117 (95.9)
Black/African American	12 (11.9)	7 (6.9)	5 (5.0)	69 (6.9)	2 (5.1)	1 (2.3)	1 (2.5)	4 (3.3)
Other^b^	1 (1.0)	0 (0.0)	2 (2.0)	9 (0.9)	0 (0.0)	0 (0.0)	1 (2.5)	1 (0.8)

Abbreviations: min–max, minimum to maximum; No. (%), number and percentage of participants in a given category; OA, older adults; RSVPreF3, prefusion conformation of the respiratory syncytial virus fusion (F) protein.

^a^The data have been published in Leroux-Roels et al [18].

^b^Includes American Indian or Alaska Native and Asian participants; 30, 60, and 120 μg AS01_E_, participants who received at least 1 dose of the AS01_E_-adjuvanted vaccine formulation with 30, 60, or 120 μg of RSVPreF3 antigen in the parent study; 30/120, 60/120, and 120/120 μg RSVPreF3-AS01_E_, participants who received 2 doses of the AS01_E_-adjuvanted vaccine formulation with 30, 60, or 120 μg of RSVPreF3 antigen in the parent study and a third dose of the AS01_E_-adjuvanted vaccine formulation containing 120 μg of RSVPreF3 antigen in the extension study (see RSVPreF3 definition); AS01_E_, adjuvant system [18].

### Safety Evaluation

**Figure 3.**
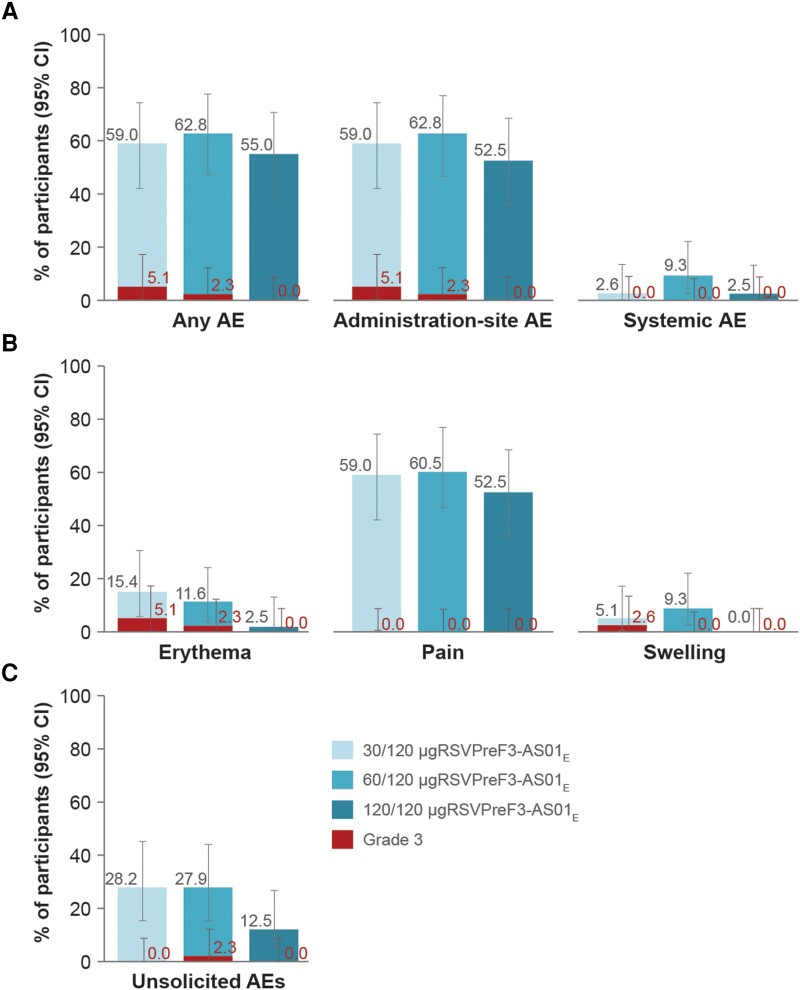
Percentage of participants reporting (*A*) at least 1 solicited AE (any, administration-site, and systemic adverse event) within 4 days, or (*B*) at least 1 solicited administration-site AE within 4 days, or (*C*) at least 1 unsolicited AE within 30 days after vaccination with the third dose of the 120 μg RSVPreF3-AS01_E_ formulation (exposed set). The only collected systemic AE was fever, which was defined as body temperature ≥38°C (grade 3 fever was defined as temperature >39°C). Grade 3 erythema and swelling were defined as being >100 mm in diameter. No serious AEs, pIMDs, and deaths were reported within 30 days after dose 3. Participants received 2 doses of the AS01_E_-adjuvanted vaccine formulation with 30, 60, or 120 μg of RSVPreF3 antigen in the parent study and a third dose of the AS01_E_-adjuvanted vaccine formulation containing 120 μg of RSVPreF3 antigen in the extension study, indicated by 30/120-, 60/120-, and 120/120 μg RSVPreF3-AS01_E_. Abbreviations: AE, adverse event; AS01_E_, adjuvant system [18]; CI, confidence interval; RSVPreF3, prefusion conformation of the respiratory syncytial virus fusion (F) protein.

Within 4 days after dose 3, solicited administration-site AEs were reported in 23 (59.0%), 27 (62.8%), and 21 (52.5%) participants in the 30/120-, 60/120-, and 120/120 μg RSVPreF3-AS01_E_ groups (Figure 3*A*). The most frequently reported solicited administration-site event was pain (in 23 [59.0%], 26 [60.5%], and 21 [52.5%] participants) (Figure 3*B*). Grade 3 administration-site erythema was reported in 2 (5.1%) and 1 (2.3%) participant in the 30/120- and 60/120 μg RSVPreF3-AS01_E_ groups, respectively, and 1 (2.6%) participant in the 30/120 μg RSVPreF3-AS01_E_ group reported grade 3 swelling. No participants in the 120/120 μg RSVPreF3-AS01_E_ group reported grade 3 administration-site AEs (Figure 3).

The only collected solicited systemic AE was fever, which was reported by 1 (2.6%) participant in the 30/120 μg RSVPreF3-AS01_E_ group, 4 (9.3%) participants in the 60/120 μg RSVPreF3-AS01_E_ group, and 1 (2.5%) participant in the 120/120 μg RSVPreF3-AS01_E_ group. No grade 3 fever (>39.0°C) was reported (Figure 3*A*).

Within 30 days after dose 3, at least 1 unsolicited AE was reported by 11 (28.2%), 12 (27.9%), and 5 (12.5%) participants in the 30/120-, 60/120-, and 120/120 μgRSVPreF3-AS01_E_ groups, respectively (Figure 3*C*). The most frequently reported unsolicited AE was headache, in 4 (10.3%; 30/120 μg RSVPreF3-AS01_E_), 1 (2.3%; 60/120 μg RSVPreF3-AS01_E_), and 1 (2.5%; 120/120 μg RSVPreF3-AS01_E_) participant. Only 1 grade 3 unsolicited AE was reported: headache in 1 (2.3%) participant in the 60/120 μg RSVPreF3-AS01_E_ group. Seven (17.9% and 16.3%) participants in each of the 30/120 and 60/120 μg RSVPreF3-AS01_E_ groups and 1 (2.5%) participant in the 120/120 μg RSVPreF3-AS01_E_ group reported at least 1 unsolicited AE considered as related to vaccination by the investigators (Supplementary Table 1). No participant reported an SAE within 30 days after vaccination.

Until end of study (6 months after dose 3), 1 (2.6%) participant in the 30/120 μg RSVPreF3-AS01_E_ group, 2 (4.7%) participants in the 60/120 μg RSVPreF3-AS01_E_ group, and 1 (2.5%) participant in the 120/120 μg RSVPreF3-AS01_E_ group reported at least 1 SAE (Supplementary Table 2). None of the SAEs were considered vaccine related by the investigators. No AEs led to withdrawal from the study, and no pIMDs or deaths were reported in this study.

### Immunogenicity Evaluation

**Figure 4.**
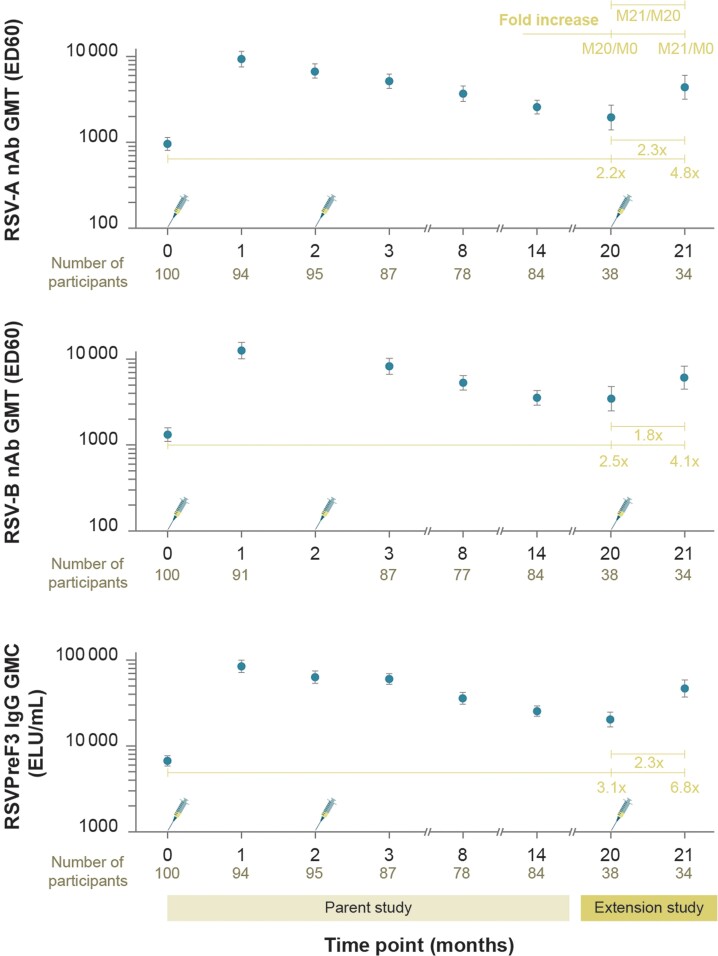
Humoral immune responses in terms of RSV-A and RSV-B nAb GMTs (ED60) and RSVPreF3-specific IgG GMCs (ELU/mL) in the 120/120 μg RSVPreF3-AS01_E_ group (per-protocol set). Part of these data (until M14) have been published in the parent study [18]; only data for 120/120 μg RSVPreF3-AS01_E_ formulation were obtained in the present (extension) study. Syringe symbols represent vaccination. Fold increase indicates fold increase in GMT and GMC values at M20 (before dose 3 in extension study) and M21 (1 month after dose 3 in the extension study) compared to M0 (before dose 1 in parent study) as well as GMT and GMC fold increase at M21 compared to M20. Time points 0, 1, 2, 3, 8, and 14 designate M0 (day 1), M1 (day 31), M2 (day 61), M3 (day 91), M8, and M14 in the parent study, respectively. Neutralizing titers against RSV-B were not measured at M2. Data are plotted as mean values with 95% confidence intervals. Participants received 2 doses of the AS01_E_-adjuvanted vaccine formulation with 120 μg of RSVPreF3 antigen in the parent study and a third dose of the AS01_E_-adjuvanted vaccine formulation containing 120 μg of RSVPreF3 antigen in the extension study, indicated by 120/120 μg RSVPreF3-AS01_E_. Abbreviations: AS01_E_, adjuvant system [18]; ED60, estimated dilution 60; ELU, enzyme-linked immunosorbent assay units; GMC/GMT, geometric mean concentration/titer; IgG, immunoglobulin G; M, month; nAb, neutralizing antibody; RSV-A and RSV-B, respiratory syncytial virus subtypes A and B; RSVPreF3, RSV fusion protein stabilized in its prefusion trimeric conformation.

The RSV-A and RSV-B nAb GMTs (ED60) at M20 were 1957.4 (95% CI, 1404.4–2728.1) and 3459.6 (95% CI, 2492.5–4801.9) (Figure 4). These observed GMT values were lower than at M14 [18], but remained higher than prevaccination (baseline, M0) (Figure 4). At M21 (1 month after dose 3), the RSV-A and RSV-B nAb GMTs were 4394.9 (95% CI, 3191.3–6052.5) and 6094.3 (95% CI, 4476.8–8296.4). The geometric mean (GM) fold increases of neutralizing titers at M21 versus M20 were 2.3 (RSV-A) and 1.8 (RSV-B) (Figure 4 and Supplementary Figure 1). Compared to baseline (M0) values, the equivalent fold increases at M1, M14, and M21 were 9.5, 2.7, and 4.8 for RSV-A and 9.2, 2.8, and 4.1 for RSV-B nAb (Figure 4 and Supplementary Figure 1) [18].

The RSVPreF3-specific IgG GMCs (ELISA units/mL) were 20 202.5 (95% CI, 16 569.5–24 632.0) at M20 and 46 276.5 (95% CI, 36 821.3–58 159.6) at M21 (Figure 4). The observed GMC values at M20 were above baseline (M0) but lower than those at M14 [18]. At M21, the RSVPreF3-specific IgG GMC was 2.3-fold higher than that at M20 (Figure 4 and Supplementary Figure 1). Compared to baseline (M0) values, the equivalent increases of IgG GMCs at M1, M14, and M21 were 12.4, 3.6, and 6.8 (Figure 4, Supplementary Figure 1) [18].

**Figure 5.**
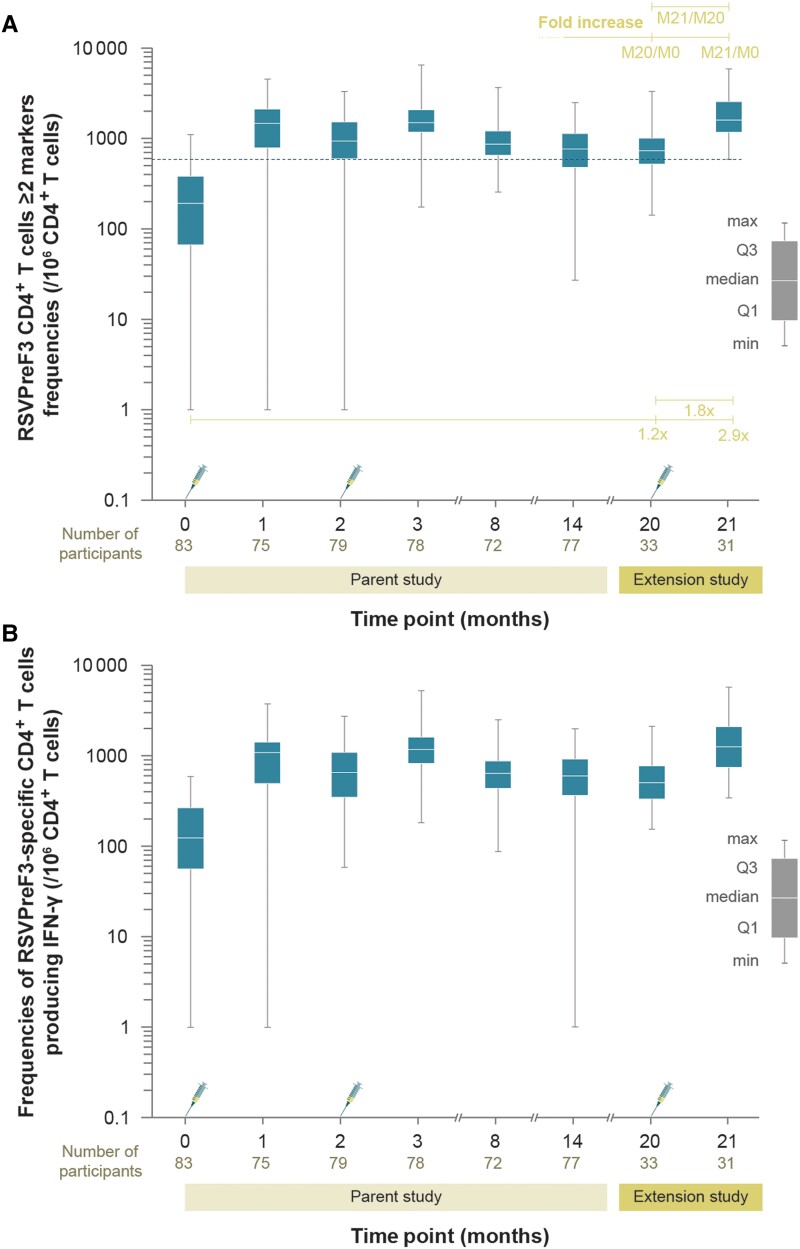
Frequencies of RSVPreF3-specific CD4^+^ T cells expressing at least (*A*) 2 markers (among IL-2, CD40L, TNF-α, IFN-γ) or (*B*) IFN-γ in the 120/120 μg RSVPreF3-AS01_E_ group (per-protocol set). Part of these data (time points to M14) have been published in the parent study [18]. Only data for 120/120 μg RSVPreF3-AS01_E_ formulation were obtained in the present (extension) study. Syringe symbols represent vaccination. The dashed horizontal line represents the assay cutoff value of 590. Fold increase and the corresponding horizontal lines indicate fold increase in frequencies of CD4^+^ T cells at M20 (before dose 3 in extension study) and M21 (1 month after dose 3 in the extension study) compared to M0 (before dose 1 in parent study), as well as fold increase in frequencies at M21 compared to M20. Time points 0, 1, 2, 3, 8, and 14 designate M0 (day 1, dose 1 vaccination), M1 (day 31, 1 month after dose 1), M2 (day 61, dose 2 vaccination), M3 (day 91, 1 month after dose 2), M8, and M14 in the parent study. Data are plotted as box and whisker plots with a median, interquartile range (Q1 and Q3, first and third quartile), minimum and maximum. Participants received 2 doses of the AS01_E_-adjuvanted vaccine formulation with 120 μg of RSVPreF3 antigen in the parent study and a third dose of the AS01_E_-adjuvanted vaccine formulation containing 120 μg of RSVPreF3 antigen in the extension study, indicated by 120/120 μg RSVPreF3-AS01_E_. Abbreviations: AS01_E_, adjuvant system [18]; CD4^+^, cluster-of-differentiation-4-expressing; CD40L, cluster of differentiation 40 ligand; IFN-γ, interferon-γ; IL-2, interleukin 2; M, month; RSVPreF3, respiratory syncytial virus fusion protein stabilized in its prefusion trimeric conformation; TNF-α, tumor necrosis factor-α.

The median frequency (per 10^6^ cells) of RSVPreF3-specific CD4^+^ T cells expressing at least 2 markers (among IL-2, CD40L, TNF-α, IFN-γ) was 731 (range, 142–3308) at M20, comparable to 764 (range, 27–2488) at M14 in the parent study (Figure 5*A*). At M21, the median frequency of these CD4^+^ T cells was 1601 (range, 589–5848), comparable to the M1 value (1466; range, 1–4593) [18]. At M21, frequencies of CD4^+^ T cells expressing at least 2 markers were 1.8- and 2.9-fold higher compared to M20 and baseline (M0), respectively (Figure 5*A* and Supplementary Figure 2). A similar profile was observed for CD4^+^ T cells producing at least IFN-γ (Figure 5*B*). Consistent with the previously reported results [18], no CD8^+^ T-cell responses were detected after vaccination with dose 3 of the 120 μg RSVPreF3-AS01_E_ formulation (Supplementary Figure 3).

## DISCUSSION

With the world population aging, disease prevention and reduced disease burden are important focus points of public health care. RSV is a common pathogen that can lead to severe respiratory disease in the OA population. OAs are susceptible to developing infection-associated morbidities and may be unable to mount an effective protective response against RSV [4, 16]. An RSV vaccine tailored toward the OA population will thus need to maximize the elicited immune responses, to overcome age-related immunosenescence, and to protect OAs against RSV-associated disease [4, 12]. Together with the ongoing phase 3 trials [30, 31], this extension study provides further insights into vaccine-induced immune responses in the OA population.

Prior to the first vaccination, the enrolled OA participants were seropositive for RSV-A and RSV-B nAb [18] due to previous exposure to RSV. Following the 2-dose vaccination in the parent study, both humoral (RSVPreF3-specific IgG GMCs, and RSV-A and RSV-B nAb GMTs) and CMI (frequencies of CD4^+^ T cells expressing at least 2 markers among IL-2, CD40L, TNF-α, and IFN-γ) responses were highest at 1 month after dose 1 (M1, day 31), without an added effect of RSVPreF3-based vaccine dose 2 (M3, day 91) [18]. These immune responses remained above baseline until 12 months after dose 2 (M14), although at lower levels than measured at M1 [18].

The described RSV-specific antibodies and CD4^+^ T cells persisted until revaccination in this study (M20), although with different kinetics. At the start of this extension study (M20), the IgG and nAb levels were lower than at M14 (parent study) but still higher than before dose 1 (M0). This is consistent with data reported for other RSV candidate vaccines [17, 24, 26, 32–36]. Importantly, however, the third 120 μg RSVPreF3-AS01_E_ dose induced an increase in RSVPreF3-specific IgG and RSV-A and RSV-B nAb levels by approximately 2-fold at M21 compared to M20. These findings demonstrate that 120 μg RSVPreF3-AS01_E_–induced antibody levels remain above baseline for at least 18 months after the second vaccination and can be increased again by administering a third vaccine dose. The observed boosting of antibodies to levels below those measured after the first vaccination appears to be a common observation in the RSV vaccine field [23, 26, 35] and, thus, not specific to the RSVPreF3-based vaccine.

The observed humoral responses were coupled with the induction of CD4^+^ T-cell immunity. An important finding was that the frequencies of CD4^+^ T cells expressing at least 2 markers did not decrease further between M14 (parent study) and M20. Additionally, as measured at M21, the CD4^+^ T-cell compartment was stimulated to the level comparable to that observed 1 month after dose 1 (M1 in the parent study). Similar to the parent study [18], the predominant T-cell response profile was CD4^+^ T-helper cells 1 (Th1; cells expressing at least IFN-γ), without detectable CD8^+^ T-cell responses. It therefore appears that the 120 μg RSVPreF3-AS01_E_ vaccine formulation induces stable CD4^+^ Th1-biased cellular immune responses, which persist for at least 18 months after the second vaccination and increase with revaccination. These findings suggest that T-cell memory induced by the primary schedule of 120 μg RSVPreF3-AS01_E_ remained boostable 18 months after dose 2 (M20). The maintenance of a CD4^+^ Th1-biased cellular immune response through vaccinations with 120 μg RSVPreF3-AS01_E_ is particularly important, as it is thought that Th1 CMI plays an important role in protecting against RSV disease [12, 14, 16].

An immunological correlate of protection for RSV is not yet established. However, the strong humoral and CMI responses elicited by the RSVPreF3 OA vaccine might be indicative of vaccine efficacy, as recently demonstrated in a phase 3 study [27, 28].

The safety findings of this extension study, in terms of solicited and unsolicited AE occurrences, are in line with previously published results [18]. Although the low number of enrolled participants was a limitation, the third dose of 120 μg RSVPreF3-AS01_E_ was well tolerated when administered 18 months after dose 2 (AS01_E_-adjuvanted, containing either 30, 60, or 120 μg of RSVPreF3). No deaths, pIMDs, nor vaccine-related SAEs were reported during the extension study period.

The limitations of this study were the relatively low number of participants and the short follow-up time. Also, because the RSVPreF3 OA vaccine has been authorized as a single dose regimen in OAs, the generalizability of the present data is limited. However, even though these data as such will not be applicable to how the vaccine is authorized for the OA population, this study provides valuable information on the safety of a booster dose, as well as on the profile and persistence of immune responses after vaccination. The ongoing phase 3 studies are currently evaluating the long-term vaccine efficacy and immune responses after different revaccination schedules with 120 μg RSVPreF3-AS01_E_ administered as a single dose in OAs [27, 28]. Study strengths include comprehensive immunogenicity evaluation and a close safety follow-up.

In conclusion, the third dose of the selected 120 μg RSVPreF3-AS01_E_ formulation administered 18 months after the second dose was well tolerated and induced an increase in both humoral and CMI responses.

## Supplementary Data

Supplementary materials are available at *The Journal of Infectious Diseases* online. Consisting of data provided by the authors to benefit the reader, the posted materials are not copyedited and are the sole responsibility of the authors, so questions or comments should be addressed to the corresponding author.

## Supplementary Material

jiad321_Supplementary_Data

## Notes

***Author contributions***. M. V. R., N. D. S., C. V., and M. P. D. contributed to the conceptualization of the study. I. L. R., M. V. R., N. D. S., B. S., C. Vm, S. K., and V. H. contributed to data collection or generation. M. V. R., N. D. S., and B. S. contributed to methodology. M. P. D., C. V. A., and S. K. contributed to formal analysis. I. L. R., M. V. R., C. V. A., N. D. S., B. S., C. V., M. P. D., S. K., and V. H. were involved in data analysis/interpretation. S. K. drafted the original draft of the manuscript. All authors contributed to manuscript review and editing and approved the final version of the manuscript.

***Acknowledgments***. We thank all study participants and their families, as well as the staff at the participating institutions. We thank staff at GSK Clinical Laboratory Sciences and GSK Clinical Department for technical assistance, in particular Anne-Marie Camier, Frederique Bossiroy, Ouafae Bouchikhi, Stephanie Howard, Geetha Kamath, Stacey Lewis, Anne Meulemans, Amoolya Modi, Maggie Schultz, Elisabeth Ngalula Kabanga, Melanie Steffens, and Jelena Tica. We also thank Yolanda Penders, Erik Jongert, and Cecile Felu for their critical review of the manuscript. Finally, we acknowledge the contribution of Frédéric Renaud, who was responsible for statistical analyses and review of the clinical study report. We also thank Akkodis Belgium for medical writing support (Irena Zurnic Bönisch), artwork design support, editorial assistance, and manuscript coordination, on behalf of GSK.

***Data availability***. Anonymized individual participant data and study documents can be requested for further research from www.clinicalstudydatarequest.com (study ID 213569). The study protocol, statistical analysis plan, and results summary are posted on ClinicalTrials.gov (NCT04657198).

***Financial support**.* This work was supported by GlaxoSmithKline Biologicals SA. GlaxoSmithKline Biologicals SA was involved in all stages of the study conduct and analysis and took responsibility for all costs associated with the development and the publishing of the article.
