# Supplementary material for: Safety and Immunogenicity of a Revaccination With a Respiratory Syncytial Virus Prefusion F Vaccine in Older Adults: A Phase 2b Study
Source: J Infect Dis. 2023 Sep 12;229(2):355–66. doi: 10.1093/infdis/jiad321 (PMC10873183; doi:10.1093/infdis/jiad321)
Supplement: jiad321_Supplementary_Data [file jiad321_supplementary_data.zip › 20230726_RSV_OA=ADJ-011_R1_SuppMat.docx]

**Supplementary information for “Safety and immunogenicity of a revaccination with a respiratory syncytial virus prefusion F candidate vaccine in older adults: a phase 2b study”**

# Supplementary Information

## Supplementary methods

### Selection of older adult participants for the extension study

At the time this extension study was initiated, the investigators contacted all older adult (OA; 60–80 years) participants from the parent study who: 1) received the AS01_E_-adjuvanted vaccine formulations (containing 30, 60, or 120 μg of the RSVPreF3 antigen) and 2) expressed a willingness to participate in follow-up studies when questioned and provided written confirmation in their informed consent form in the parent study. Of these potentially eligible OA participants, only those who confirmed their willingness to participate in the extension study at the point of contact were enrolled.

### Study inclusion criteria

The participants had to meet all of the following criteria to be included in the study:

- Male or female participants who received two doses of the AS01_E_-adjuvanted formulations containing 30, 60, or 120 μg of recombinant RSVPreF3 antigen in the parent study.
- Participants who, in the opinion of the investigator, complied with the requirements of the protocol (e.g., completion of the diary cards, return for the follow-up visit, available for contact).
- Written informed consent obtained from the participant prior to performance of any study-specific procedure.

### Study exclusion criteria

• Significant underlying illness or administered therapy that, in the opinion of the investigator, would be expected to prevent participation in the study.

• Any confirmed or suspected immunosuppressive or immunodeficient condition based on information on concomitant medication/vaccination collected prior to the study start and physical examination (no laboratory testing required).

• Serious or unstable chronic illness that developed during or after the parent study. Patients with chronic stable medical conditions with or without specific treatment, such as diabetes, hypertension, or cardiac disease, were allowed to participate in this study if considered by the investigator as clinically stable.

• Recurrent or un-controlled neurological disorders or seizures that developed during or after the parent study. Participants with medically controlled active or chronic neurological diseases could be enrolled in the study as per-investigator assessment, provided that their condition allowed them to comply with the requirements of the protocol (e.g., completion of diary cards, attend phone calls, study site visits).

• Significant underlying illness that developed during or after the parent study and that, in the opinion of the investigator, would be expected to prevent completion of the study (e.g., life-threatening disease likely to limit survival to less than 6 months).

• Lymphoproliferative disorder and malignancy developed during or after the parent study.

• Any medical condition that developed during or after the parent study and that, in the judgement of the investigator, would make intramuscular injection unsafe.

• Previous vaccination with an RSV vaccine, other than the one in the parent study.

• Use of any investigational or non-registered product (e.g., drug, vaccine, or medical device) other than the study vaccine during the period beginning 30 days before the dose of study vaccine, or planned use during the study period.

• Planned or actual administration of a vaccine not foreseen by the study protocol in the period starting 30 days before and ending 30 days after the dose of study vaccine administration, with the exception of inactivated, split virion and subunit influenza vaccines which can be administered up to 14 days before or from 30 days after the study vaccination.

Note: In case an emergency mass vaccination for an unforeseen public health threat (e.g., a pandemic) is recommended and/or organised by the public health authorities, outside the routine immunisation programme, the time period described above could be reduced if necessary for that vaccine provided it was used according to the local governmental recommendations and provided that the Sponsor was notified accordingly.

• Administration of long-acting immune-modifying drugs or planned administration at any time during the study period (e.g., infliximab).

• Administration of immunoglobulins and/or any blood products or plasma derivatives during the period starting 90 days before the dose of study vaccine, or planned administration during the study period.

• Chronic administration (defined as more than 14 consecutive days in total) of immunosuppressants or other immune-modifying drugs during the period starting 90 days prior to the vaccine dose, or planned administration during the study period. For corticosteroids, this meant prednisone ≥20 mg/day, or equivalent. Inhaled and topical steroids were allowed.

• Confirmed use or anticipated use of immunosuppressive/cytotoxic therapy (e.g., medication used during cancer chemotherapy, organ transplantation, or to treat autoimmune disorders).

• Concurrently participating in another clinical study, at any time during the study period, in which the participant was exposed to an investigational or a non-investigational vaccine/product (pharmaceutical product or invasive medical device).

• Bedridden participants.

• Planned move to a location that prohibited participating in the trial.

• History of chronic alcohol consumption and/or drug abuse developed during or after the parent study as deemed by the investigator to render the potential participant unable/unlikely to provide accurate safety reports or comply with study procedures.

### Definition of analysis sets

The exposed set (ES) was used primarily for safety and demography analyses. ES included all participants who received at least one dose of the investigational vaccine or placebo (parent study) or dose 3 of the RSVPreF3 OA vaccine (extension study). Immunogenicity analyses were conducted on the per-protocol set (PPS). In both the parent and extension studies, the PPS included all participants meeting the eligibility criteria, who were vaccinated according to protocol, did not present with any conditions, did not receive any concomitant treatments that would exclude them from the study, who complied with the vaccination schedule, and provided valid samples for immunogenicity analyses at the indicated timepoints.

### Tertiary objectives and endpoints

- The tertiary objective was to further characterise RSVPreF3-vaccine immunogenicity. The specific endpoint was to assess frequencies of cluster-of-differentiation-4-expressing (CD4+) and CD8+ T-cells expressing one or any combination of immune marker(s) in vitro until one month post-dose 3 (M21).

**Supplementary table 1**: Number and percentage of participants with at least one vaccine-related (as per-investigator assessment) unsolicited adverse event within 30 days post-dose 3 (Exposed set)

|  | **30/120 μg RSVPreF3-AS01_E_** | | **60/120 μg RSVPreF3-AS01_E_** | | **120/120 μg RSVPreF3-AS01_E_** | |
| --- | --- | --- | --- | --- | --- | --- |
| Primary system organ class | n | % (95% CI) | n | % (95% CI) | n | % (95% CI) |
| Any AE | 7 | 17.9 (7.5–33.5) | 7 | 16.3 (6.8–30.7) | 1 | 2.5 (0.1–13.2) |
| General disorders and administration-site conditions | 5 | 12.8 (4.3–27.4) | 6 | 14.0 (5.3–27.9) | 0 | 0.0 (0.0–8.8) |
| Chills | 2 | 5.1 (0.6–17.3) | 2 | 4.7 (0.6–15.8) | 0 | 0.0 (0.0–8.8) |
| Fatigue | 0 | 0.0 (0.0–9.0) | 3 | 7.0 (1.5–19.1) | 0 | 0.0 (0.0–8.8) |
| Influenza-like illness | 2 | 5.1 (0.6–17.3) | 1 | 2.3 (0.1–12.3) | 0 | 0.0 (0.0–8.8) |
| Malaise | 1 | 2.6 (0.1–13.5) | 1 | 2.3 (0.1–12.3) | 0 | 0.0 (0.0–8.8) |
| Axillary pain | 1 | 2.6 (0.1–13.5) | 0 | 0.0 (0.0–8.2) | 0 | 0.0 (0.0–8.8) |
| Injection-site warmth | 1 | 2.6 (0.1–13.5) | 0 | 0.0 (0.0–8.2) | 0 | 0.0 (0.0–8.8) |
| Nervous system disorders | 4 | 10.3 (2.9–24.2) | 1 | 2.3 (0.1–12.3) | 1 | 2.5 (0.1–13.2) |
| Headache | 4 | 10.3 (2.9–24.2) | 1 | 2.3 (0.1–12.3) | 1 | 2.5 (0.1–13.2) |
| Paraesthesia | 1 | 2.6 (0.1–13.5) | 0 | 0.0 (0.0–8.2) | 0 | 0.0 (0.0–8.8) |
| Gastrointestinal disorders | 1 | 2.6 (0.1–13.5) | 0 | 0.0 (0.0–8.2) | 0 | 0.0 (0.0–8.8) |
| Diarrhoea | 1 | 2.6 (0.1–13.5) | 0 | 0.0 (0.0–8.2) | 0 | 0.0 (0.0–8.8) |
| Infections and infestations | 0 | 0.0 (0.0–9.0) | 1 | 2.3 (0.1–12.3) | 0 | 0.0 (0.0–8.8) |
| Oral herpes | 0 | 0.0 (0.0–9.0) | 1 | 2.3 (0.1–12.3) | 0 | 0.0 (0.0–8.8) |
| Musculoskeletal and connective tissue disorders | 0 | 0.0 (0.0–9.0) | 1 | 2.3 (0.1–12.3) | 0 | 0.0 (0.0–8.8) |
| Arthralgia | 0 | 0.0 (0.0–9.0) | 1 | 2.3 (0.1–12.3) | 0 | 0.0 (0.0–8.8) |
| Myalgia | 0 | 0.0 (0.0–9.0) | 1 | 2.3 (0.1–12.3) | 0 | 0.0 (0.0–8.8) |
| Respiratory, thoracic, and mediastinal disorders | 1 | 2.6 (0.1–13.5) | 0 | 0.0 (0.0–8.2) | 0 | 0.0 (0.0–8.8) |
| Increased upper airway secretion | 1 | 2.6 (0.1–13.5) | 0 | 0.0 (0.0–8.2) | 0 | 0.0 (0.0–8.8) |
| 30/120, 60/120, and 120/120 μg RSVPreF3-AS01_E_, participants who received two doses of the AS01_E_-adjuvanted vaccine formulation with 30, 60, or 120 μg of RSVPreF3 antigen in the parent study and a third dose of the AS01_E_-adjuvanted vaccine formulation containing 120 μg of RSVPreF3 antigen in the extension study (see RSVPreF3 definition further on); % (95% CI), percentage of participants with a 95% confidence interval in a given category; AE, adverse event; AS01_E_, adjuvant system [18]; n, number of participants in a given category; RSVPreF3, RSV fusion protein stabilised in its trimeric prefusion conformation. | | | | | | |

**Supplementary table 2**: Listings of participants who reported at least one serious adverse event up to six months post-dose 3 (Exposed set)^a^

| Group (μg RSVPreF3-AS01_E_) | Gender | Age^b^ | Event | Onset (days)^c^ | Duration (days) | Intensity | Medical visit | Outcome |
| --- | --- | --- | --- | --- | --- | --- | --- | --- |
| 30/120 | Male | 64 | Diverticulitis | 123 | 14 | Severe | Hospitalisation | Resolved |
| 60/120 | Male | 63 | Limb traumatic amputation | 43 | 126 | Severe | Hospitalisation | Resolved, with sequelae |
|  | Male | 64 | Benign prostatic hyperplasia | 105 | 109 | Severe | Hospitalisation | Resolved, with sequelae |
| 120/120 | Male | 74 | Prostate cancer | 71 | N/A | Mild | Hospitalisation | Resolving^d^ |
| ^a^No SAEs were considered related to the study vaccine, as per-investigator assessment; ^b^Age at onset of an adverse event; ^c^Number of days post-dose 3; ^d^Event was not resolved at the time of study report; 30/120-, 60/120-, and 120/120, participants who received two doses of the AS01_E_-adjuvanted vaccine formulation with 30, 60, or 120 μg of RSVPreF3 antigen in the parent study and a third dose of the AS01_E_-adjuvanted vaccine formulation containing 120 μg of RSVPreF3 antigen in the extension study (see RSVPreF3 definition further on); AS01_E_, adjuvanted system [18]; N/A, not applicable; RSVPreF3, RSV fusion protein stabilised in its trimeric prefusion conformation; SAE, serious adverse event. | | | | | | | | |

**Supplementary figure 1**: Geometric mean of the fold increase of neutralising titres against RSV-A and RSV-B (ED60) and RSVPreF3-specific IgG antibody concentrations (ELU/mL) post-vaccination compared to pre-vaccination in the 120/120 μg RSVPreF3-AS01_E_ group (Per-protocol set)^a–d^

^a^Part of these data (until Month 14 [M14]) have already been published in the parent study [18]; ^b^Only data for 120/120 μg RSVPreF3-AS01_E_ formulation were obtained in the present (extension) study; ^c^Syringe symbols represent vaccination; ^d^Data points are not shown for Day 1 (M0) because folds increase were calculated relative to Day 1; ^e^Timepoints 0, 1, 2, 3, 8, and 14 designate M0 (Day 1), M1 (Day 31), M2 (Day 61), M3 (Day 91), M8, and M14 in the parent study, respectively. Data are plotted as mean values with 95% confidence intervals. 120/120 μg RSVPreF3-AS01_E_, participants who received two doses of the AS01_E_-adjuvanted vaccine formulation with 120 μg of RSVPreF3 antigen in the parent study and a third dose of the AS01_E_-adjuvanted vaccine formulation containing 120 μg of RSVPreF3 antigen in the extension study (see RSVPreF3 definition further on); AS01_E_, adjuvant system [18]; ED60, estimated dilution 60; ELU, enzyme-linked immunosorbent assay units; GM, geometric mean; GMC and GMT, GM concentrations and titres; IgG, immunoglobulin G; nAb, neutralising antibody; RSV-A and RSV-B, respiratory syncytial virus subtypes A and B; RSVPreF3, RSV fusion protein stabilised in its prefusion trimeric conformation. **Supplementary figure 2**: Fold increase of the frequency of RSVPreF3-specific CD4+ T-cells (per million of CD4+ T-cells) expressing at least two markers (IL-2, CD40L, TNF-α, IFN-γ) in the 120/120 μg RSVPreF3-AS01_E_ group (Per-protocol set)^a–d^

^a^Part of these data (until Month 14 [M14]) have already been published in the parent study [18]; ^b^Only data for 120/120 μg RSVPreF3-AS01_E_ formulation were obtained in the present (extension) study; ^c^Syringe symbols represent vaccination; ^d^Data points are not shown for Day 1 (M0) because folds increase were calculated relative to Day 1; ^e^Timepoints 0, 1, 2, 3, 8, and 14 designate M0 (Day 1), M1 (Day 31), M2 (Day 61), M3 (Day 91), M8, and M14 in the parent study, respectively. Data are plotted as median values with minimum and maximum. 120/120 μg RSVPreF3-AS01_E_, participants who received two doses of the AS01_E_-adjuvanted vaccine formulation with 120 μg of RSVPreF3 antigen in the parent study and a third dose of the AS01_E_-adjuvanted vaccine formulation containing 120 μg of RSVPreF3 antigen in the extension study (see RSVPreF3 definition further on); AS01_E_, adjuvant system [18]; CD4+, cluster-of-differentiation-4-expressing; CD40L, cluster of differentiation 40 ligand; IFN-γ, interferon gamma; IL-2, interleukin 2; RSVPreF3, respiratory syncytial virus fusion (F) protein stabilised in its prefusion trimeric conformation; TNF-α, tumour necrosis factor alpha.

**Supplementary figure 3**: Frequency of RSVPreF3-specific-CD8+ T-cells expressing at least two markers among IL-2, CD40L, TNF-α and IFN-γ (per million CD8+ T-cells) (Per-protocol set)^a^

^a^Only data for 120/120 μg RSVPreF3-AS01_E_ formulation were obtained in the present (extension) study. Data are plotted as median values with interquartile range (Q1 and Q3 representing the first and third quartile), minimum and maximum. 120/120 μg RSVPreF3-AS01_E_, participants who received two doses of the AS01_E_-adjuvanted vaccine formulation with 120 μg of RSVPreF3 antigen in the parent study and a third dose of the AS01_E_-adjuvanted vaccine formulation containing 120 μg of RSVPreF3 antigen in the extension study (see RSVPreF3 definition further on); AS01_E_, adjuvant system [18]; CD8+, cluster-of-differentiation-8-expressing; CD40L, cluster of differentiation 40 ligand; IFN-γ, interferon gamma; IL-2, interleukin 2; RSVPreF3, respiratory syncytial virus fusion (F) protein stabilised in its prefusion trimeric conformation; TNF-α, tumour necrosis factor alpha.
