# Supplementary figures and images for: Safety and Immunogenicity of a Revaccination With a Respiratory Syncytial Virus Prefusion F Vaccine in Older Adults: A Phase 2b Study
Source: J Infect Dis. 2023 Sep 12;229(2):355–66. doi: 10.1093/infdis/jiad321 (PMC10873183; doi:10.1093/infdis/jiad321)

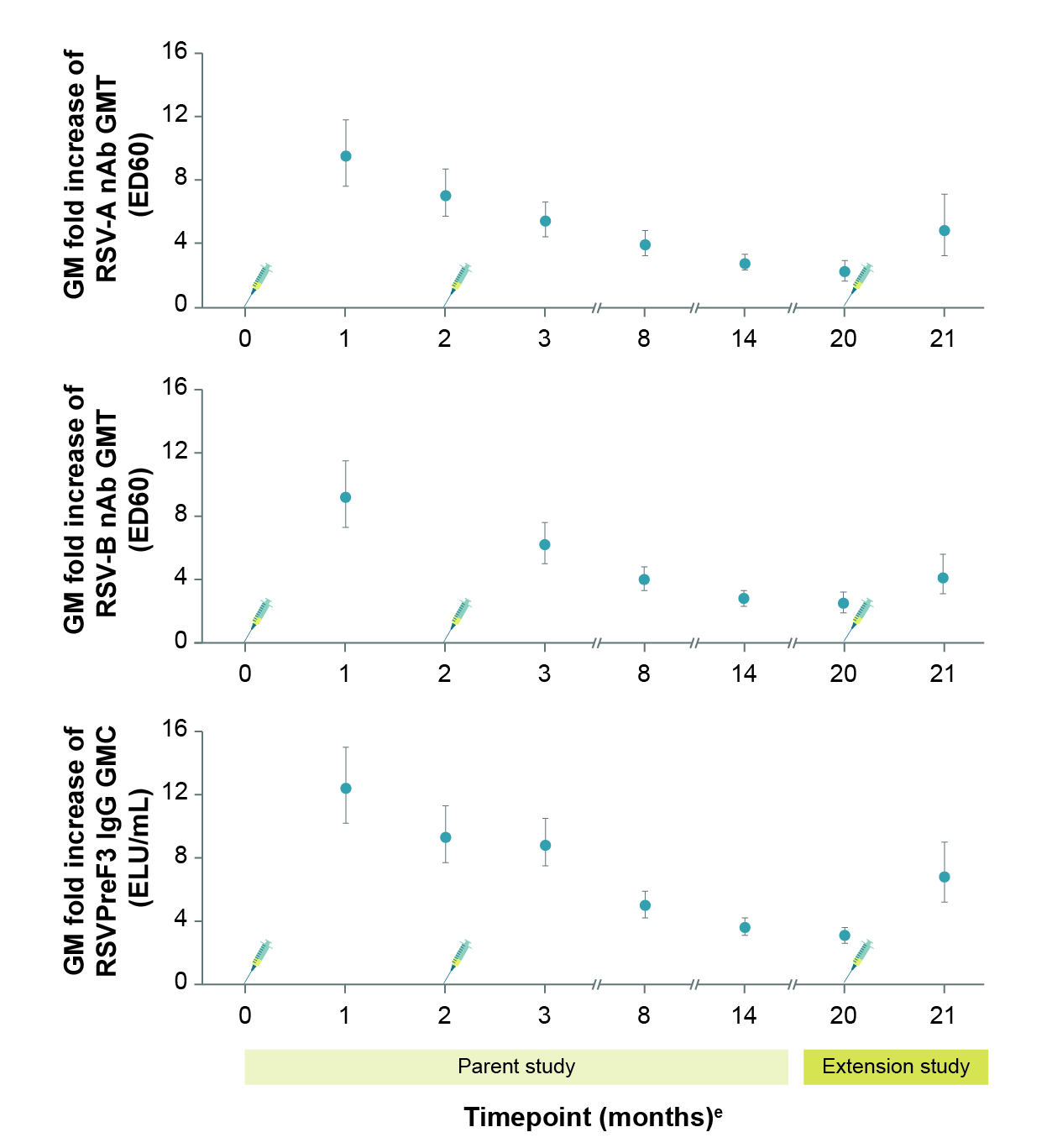

Supplement: jiad321_Supplementary_Data [file jiad321_supplementary_data.zip › RSV OA 011 Suppl fig 1.tif]

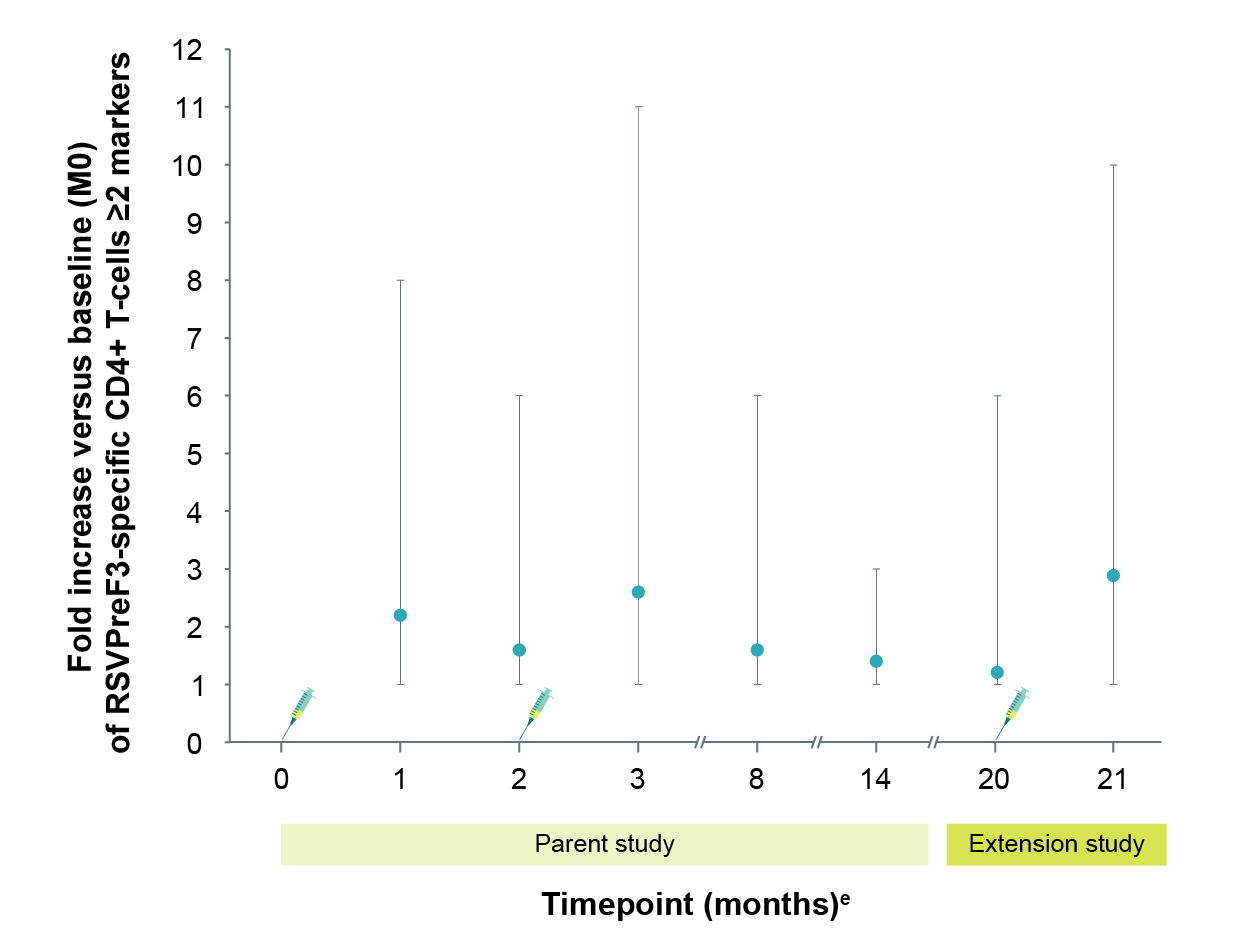

Supplement: jiad321_Supplementary_Data [file jiad321_supplementary_data.zip › RSV OA 011 Suppl fig 2.tif]

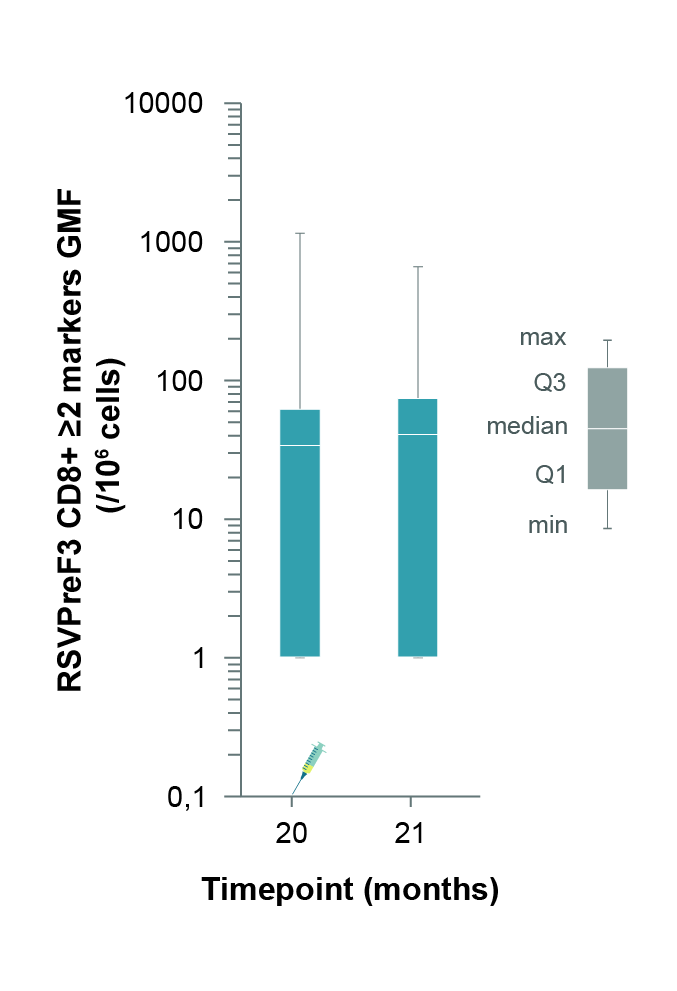

Supplement: jiad321_Supplementary_Data [file jiad321_supplementary_data.zip › RSV OA 011 Suppl fig 3.tif]
